# Morphological Relation of Peripheral Nerve Sheath Tumors and Nerve Fascicles: Prospective Study and Classification

**DOI:** 10.3390/jcm11030552

**Published:** 2022-01-22

**Authors:** Matthias Holzbauer, Kathrin Aufschnaiter-Hießböck, Maximilian Zaussinger, Oskar C. Aszmann, Manfred Schmidt

**Affiliations:** 1 Section of Plastic and Reconstructive Surgery, Department of General Surgery, Med Campus III, Kepler University Hospital Linz, Krankenhausstrasse 9, 4020 Linz, Austria; matthias.holzbauer@a1.net (M.H.); m.zaussinger@hotmail.com (M.Z.); 2 Faculty of Medicine, Johannes Kepler University Linz, Altenbergerstraße 69, 4020 Linz, Austria; Kathrin.aufschnaiter-hiessboeck@keplerklinikum.at; 3 Microsurgical Training and Research Center (MAZ), Kepler University Hospital GmbH, Krankenhausstrasse 9, 4020 Linz, Austria; 4 Department of Neurosurgery, Neuro Med Campus, Kepler University Hospital Linz, Wagner-Jauregg-Weg 15, 4020 Linz, Austria; 5 Department of Plastic and Reconstructive Surgery, Medical University of Vienna, Währinger Gürtel 18-20, 1090 Vienna, Austria; oskar.aszmann@meduniwien.ac.at

**Keywords:** benign peripheral nerve sheath tumor, classification, neurofibroma, schwannoma, tumor enucleation

## Abstract

Removal of benign peripheral nerve sheath tumors (bPNST) represents a surgical challenge. The morphological relation of bPNST and healthy nerve fascicles are of utmost importance for achieving both removal of the entire tumor and preservation of functional integrity of the peripheral nerve. Thus, we intraoperatively assessed the morphological patterns between bPNST and nerve fascicles using photo documentation obtained between January 2009 and September 2021. In 31 patients (20 women and 11 men) with a mean age of 48 ± 18 years a total of 34 bPNST were removed. Four constant morphological patterns between bPNST relatively to nerve fascicles were detected: (1) bPNST is located peripherally (*n* = 16), (2) it splits the nerve into two main fascicles (*n* = 5), (3) it totally splits up the nerve out of the nerve’s center (*n* = 8) und (4) it encloses the nerve and its fascicles (*n* = 5) without any detectable boundary layer. Histology revealed 28 schwannomas, five neurofibromas, and one perineurioma. The proposed classification reflects the increasing complexity of tumor removal with a higher type number. This might be beneficial for preoperative diagnostics, i.e., high-resolution ultrasound or MRI-tractography, as well as for planning the bPNST’s surgical resection and the possible need for nerve reconstruction.

## 1. Introduction

Benign peripheral nerve sheath tumors (bPNST) constitute 10–12% of all benign soft-tissue neoplasms [1,2]. Neurofibromas, schwannomas and perineuriomas represent the common, major categories of bPNST, which were historically defined according to the cell types involved [3]: while the last two entities consist of a uniform cell population, i.e., Schwann cells and perineurial cells, respectively, neurofibromas are composed of different cell types including fibroblasts, Schwann cells, perineurial cells and scattered axons [3,4]. Recent advances in diagnostic work-up of bPNST specimens added immunohistochemical markers and genetic analyses to conventional histology [3,5,6]. Thus, not only subtypes of the well-known schwannoma, neurofibroma, and perineurioma could be detected [3], but also hybrid nerve sheath tumors were defined as an independent category in the World Health Organization Classification of Tumors of the Central Nervous System [3].

Clinically, most patients with a symptomatic bPNST present with pain and/or a palpable soft tissue mass [2]. Symptoms of a mononeuropathy including numbness, weakness, or paresthesia occur in less than 20% of patients with this condition [2]. Generally, bPNST occur either solitarily or due to neurofibromatosis as underlying disease, which can be genetically further divided into Neurofibromatosis Type 1, 2, and Schwannomatosis [7]. However, the stepwise diagnostic approach for symptomatic peripheral nerve sheath tumors (PNST) is principally the same: it includes clinical examination, ultrasound investigation and high-resolution MRI. MRI scans have shown to provide a good sensitivity and specificity to differentiate between benign and malignant PNST, which are associated with a very poor survival rate. This radiologic work-up is also used for planning the gold-standard therapy for symptomatic bPNST, i.e., surgical resection. However, correct delineation of the tumor and healthy nerve fascicles based on these images renders a difficult task.

First and foremost, the primary objective of the surgical procedure is to improve symptoms via entirely resecting the tumor whilst preserving the functional integrity of the nerve. Historically, en bloc resection of the entire nerve following graft reconstruction represented the standard therapy in benign neoplastic nerve surgery [8,9]. Considering that bPNST are slow-growing tumors which commonly do not cause neurological deficits, iatrogenic nerve injury must be imperatively avoided [10,11]. In this regard, benign neoplastic nerve surgery has evolved to microsurgical dissection and resection of bPNST. In this regard, Russel posed the “preserve the nerve” principle, in which he emphasized that a fascicle-sparing enucleation of the lesion should always be the primary approach when performing bPNST resection [12]. Only if no dissection layer between bPNST and nerve fascicles can be identified, the more invasive, historical method including *en bloc* resection and nerve reconstruction is still used. Therefore, the intraoperative morphological relation between the bPNST and healthy nerve fascicles is the crucial factor in determining which surgical technique is feasible and how technically demanding this procedure will be. Thus, the present study aimed to identify any morphological patterns between bPNST and healthy nerve fascicles.

## 2. Materials and Methods

Between January 2009 and September 2021, all patients presenting with a PNST were included in the present prospective study. Preoperative imaging aided in planning the surgical procedure and to differentiate between malignant entities [13,14].

Inclusion criteria involved patients at any age and a bPNST located at any peripheral nerve. Eligibility for inclusion required proper, intraoperative photographs of the initial exposure and during the resection of the tumor. Moreover, the operative report describing the position of the bPNST relatively to the physiological nerve and containing the performed surgical technique for resection as well as histological work-up had to be available.

We defined revision surgery for PNST as exclusion criteria. Furthermore, if histological examination did not confirm the tumorous lesion to be a bPNST, patients were also excluded from this study.

The last author (MS) enrolled all patients which were eligible to participate in the present study. Written informed consent was obtained by all participants. This trial was designed as prospective case series. The present study was approved by the Institutional Review Board of Ethical Commission of Johannes Kepler University Linz (Approval Number: 1203/2021) and adheres to the WMA Declaration of Helsinki.

### 2.1. Surgical Technique

During surgery the bPNST and affected nerve were exposed in their full circumference and a few centimeters proximally and distally to the lesion. Thus, the bPNST could be rotated along the axis of the nerve. Following, electrical mapping of the tumor surface using nerve stimulation and EMG recordings was performed.

Visible nerve fascicles were noted, and intraoperative photographs were taken. Under the microscope, the area with the least number of nerve fascicles was chosen for perineural incision of the lesion parallel to the nerve fascicles. Microsurgical, blunt dissection was continued aiming to identify any capsule of the bPNST. Whenever this could be achieved, the incision was extended to both poles of the bPNST where the conglomerate of the tumor and nerve tapers into physiological nerve structures. After the parent fascicle could be identified, blunt dissection was performed along a reliable plain, i.e., the tumor capsule, to free the bPNST under gentle retraction of the surrounding nerve fascicles. As a result, the bPNST could be separated from the nerve in its full circumference while maximally preserving the functional integrity of the nerve. Afterwards, in case of a present parent fascicle, the single fascicle was carefully separated from uninvolved fascicles and consequently coagulated and transected at both poles. The operative site was again recorded via photographs.

If no plane between the bPNST and the physiological nerve could be identified for performing an enucleation of the lesion, an en bloc resection of the lesion had to be performed. Thus, the affected nerve was transected perpendicular to the long axis of the nerve in a tumor-free region located next to the bPNST. The bPNST was removed and an autologous nerve transplantation using a sural nerve graft was performed using microsurgical nerve coaptation.

### 2.2. Assessment

Demographical data containing patient’s age at time of surgery and sex were noted. Moreover, the affected nerve was registered. The intraoperative photographic documentation and the operative report of every tumor resection were screened to identify patterns in the morphological location of the bPNST relatively to healthy fascicles. After certain types were defined, the lead author and the last author independently categorized every bPNST according to the previously defined types. If any tumor was not allocated to the same type by the two examiners, this particular case was discussed in the author team to find any consensus. Afterwards, histological entities of the tumor in each group were displayed using a contingency table.

### 2.3. Statistical Methods

Data are presented using descriptive statistics. The parameter *age* was either presented as mean (±standard deviation) or as median (interquartile range (IQR)) depending on the normal distribution after the Kolmogorov-Smirnov-Test was performed. The simple frequencies of the affected nerves were given. Different types of morphological relation between bPNST and nerve fascicles are presented in a contingency table with their respective histological entity.

## 3. Results

Thirty seven PNST of 34 patients were initially included in the present study. Three cases had to be excluded because histological work-up did not confirm the tumor to be a bPNST, but rather showed an angioleiomyoma, a leiomyosarcoma, and a malignant neurofibroma, respectively. Thus, 34 bPNST were assessed in the present case series. Demographical data of our patients are displayed in Table 1. The following nerves were affected by bPNST: brachial plexus (*n* = 7), tibial nerve (*n* = 4), ulnar nerve (*n* = 4), common peroneal nerve (*n* = 3), profound peroneal nerve (*n* = 3), radial nerve (*n* = 3), sciatic nerve (*n* = 3), median nerve (*n* = 2), frontal nerve (*n* = 1), musculocutaneous nerve (*n* = 1), femoral nerve (*n* = 1), pudendal nerve (*n* = 1), and intercostal nerve (*n* = 1).

**Figure 1:**
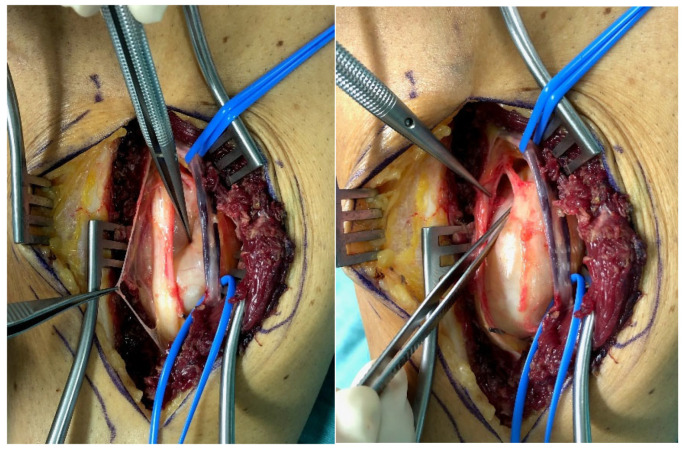
Type I lesion: Intraoperative photograph of a male patient depicts a schwannoma of the tibial nerve (**left**). The entire tumor mass was located ventrally to nerve after finishing dissection (**right**).

**Figure 2:**
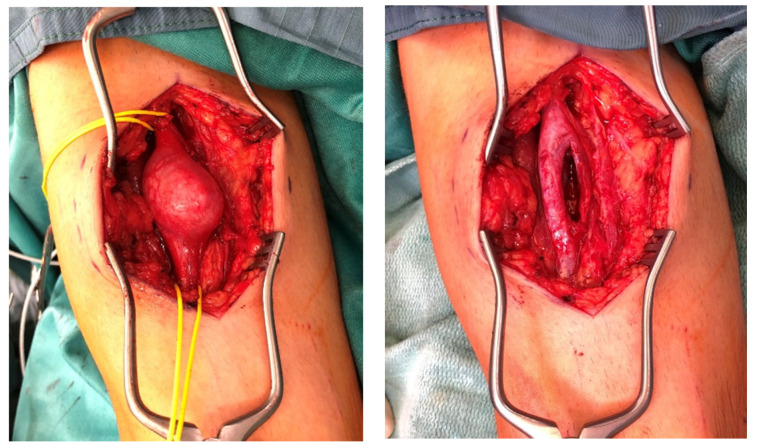
Type II lesion: A male patient intraoperatively showed a schwannoma in the upper arm region of the median nerve (**left**). After tumor removal, it can be seen that the tumor had split the median nerve into 2 main fascicles (**right**).

**Figure 3:**
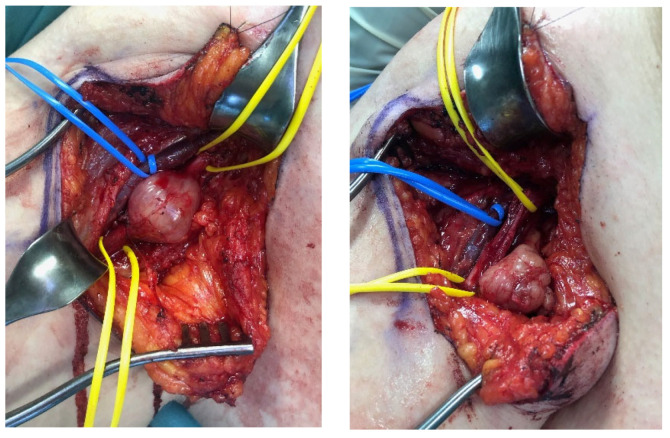
Type III lesion: Intraoperative photograph of a female patient reveals a schwannoma of the ulnar nerve in the axillar region (**left**). The tumor was located at the center of the nerve and had completely split up the nerve. After tumor resection (tumor mass was placed besides the ulnar nerve in the wound cavity), the cave which was created by the space-consuming tumor collapsed, and the nerve regained its original diameter (**right**).

**Figure 4:**
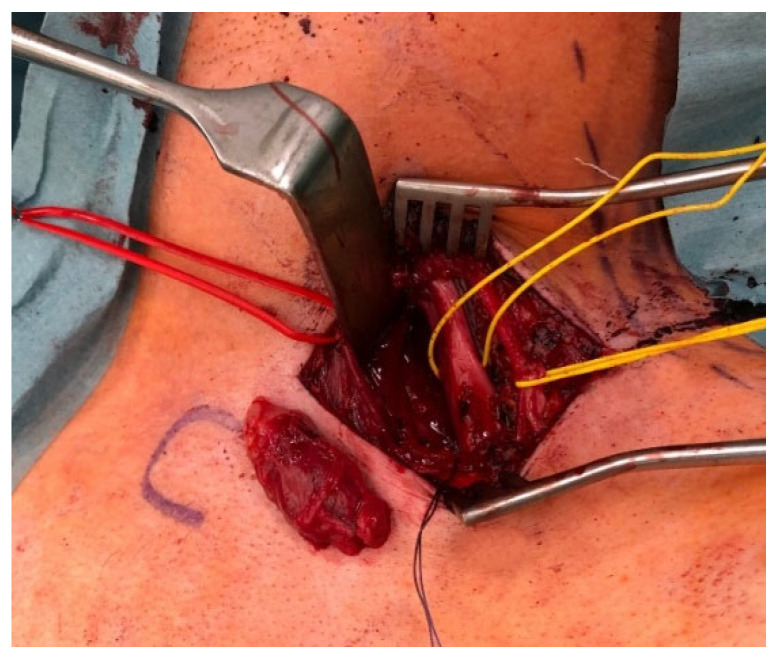
Type IV: A male patient intraoperatively presented a neurofibroma of the C7 root of the brachial plexus. The photograph shows the intraoperative situs after en bloc resection of the tumor, which was placed on the left side of the surgical approach.

Four constant morphological relation patterns between bPNST relatively to nerve fascicles were detected. Table 2 introduces these four types via textual description, and schematic illustration. Clinical examples of each type are presented in Figure 1, Figure 2, Figure 3 and Figure 4. The number of cases in our study cohort according to this classification and their respective histological entities are displayed in Table 3.

## 4. Discussion

In the present study, we were able to detect four different types regarding the morphological relation of bPNST and healthy nerve fascicles (see Table 2). Based on these results, we propose this classification for intraoperative description and documentation of the bPNST and its removal. More importantly, this classification can be used preoperatively in combination with high-resolution imaging: Schmidt et al. reported that diffusion tensor tractography represents a valuable method to visualize the relation between bPNST and healthy nerve fascicles [15]. Moreover, in selected cases of superficially located bPNST high-resolution ultrasound aids to delineate between these two structures [16]. Preoperative evaluation of the bPNST’s location using this classification enables the surgeon to estimate the complexity of surgical removal because the surgical demand as well as the risk for postoperative neurological morbidity increases with a higher type number. As legal aspects become more and more important in clinical everyday life, the extent of informed consent for surgery can be derived from the estimated type of the bPNST: Type IV inevitably requires informing the patient about a potential need for nerve transplantation and about a higher risk for postoperative neurological morbidity after tumor removal. While the previous medical literature contains one publication [17] differentiating between peripheral and central lesions, this study is—to the best of our knowledge—the first one focusing on the morphological relation between bPNST and nerve fascicles.

The rationale to perform any surgical treatment for a certain disease is always dependent and guided from the current understanding of this disease [8]. In 1855, Syme reported in an article published in The Lancet that he successfully treated a peripheral nerve tumor with amputation of the affected limb because it was “too intimately connected with the surrounding parts” [8,18]. It was not until 1969 that histological investigations of PNST revealed two different tumor entities, i.e., schwannomas and neurofibromas [8,19]. Since this distinction surgical treatment commonly varied between these two main tumor entities in the 1970s and 1980s: en-bloc resection with consecutive nerve reconstruction was the standard therapy for neurofibromas [20,21,22], while schwannomas were dissected and enucleated. The main reason for this differentiation is that more or larger parent fascicles are entering and exiting neurofibromas compared to schwannomas [12,22]. Moreover, neurofibromas were considered to be generally unencapsulated and even invasive, so that authors even warned against attempted enucleation [23]. Since then, Donnerer et al. were the first authors who advocated an equal treatment for bPNST: schwannomas as well as neurofibromas should be considered as encapsulated, removable lesions whose resection implies an acceptable risk of injury to the nerve [22]. Using the proposed classification, this risk might become predictable. Even though microsurgical techniques have improved, en bloc resection of bPNST, especially neurofibromas, continued [9,24]. Thus, Russell postulated the “*preserve the nerve*” principle for surgical resections of bPNST [12] so that iatrogenic transection of healthy nerve fascicles with consecutive neurological morbidity is no longer going to be performed. Despite different histological entities, with which the differentiation in surgical therapy was justified in past days, recent studies found even more common features of these two major tumor categories: both tumors usually have a true capsule and a pseudocapsule [25]. The differentiation between these layers is important because the pseudocapsule contains both epineural vessels and functional en passent fascicles. Assessing intraoperative photographs, Stone et al. found that both schwannomas and neurofibromas showed a yellow true capsule, which can aid identifying the proper plane for dissection. Moreover, both entities tend to rather displace than invade adjacent nerve fascicles [11,26]. Although more or larger parent fascicles are generally entering neurofibromas, theses parent fascicles are considered to be non-functional in schwannomas as well as in neurofibromas [11,25,26]. Even if neurofibromatosis represents the underlying disease for a symptomatic bPNST, the precise genetic subtype with certain immunohistochemical markers currently does not have an impact on the principal mode of treatment, i.e., surgical resection, because targeted therapies are either not available or just tested in clinical trials [7].

A limitation of the present study might be that the study cohort contains a larger number of patients with a schwannoma compared to the ones with a neurofibroma. Nevertheless, our results corroborate the beforementioned findings that the histological entity of the tumor has no impact on the complexity of surgical resection, because type IV lesions revealed nearly the same number of neurofibromas and schwannomas in the histological work-up. Apart from that, nearly half of our neurofibroma cases prove to be type I; hence, implying relatively low surgical complexity and risk for neurological morbidity. Furthermore, another potential limitation might be that our histological analysis detected major entities but no subtypes. Regarding the definition of type IV (*bPNST encloses nerve fascicles*) it must be added that other authors also found some lesions to be more adherent to the surrounding pseudocapsule, which rendered the identification of the boundary layer difficult [25,27]. Even though bPNST showed a low recurrence rate of 3.8% in a large literature review [28], subtotal resection resulted in a higher recurrence rate [28]. Moreover, subtotal resection in combination with the failure to achieve enucleation due to difficulties to find the proper dissection plain also resulted in a higher rate of neurological morbidity [17,29]. In case of type IV lesions, general considerations in bPNST removal, i.e., the balance between aiming a gross total resection and risking neurological injury, become even more important, especially because potential revision surgery is associated with functional loss due to scarring [26].

In a further step of this study, we are planning to correlate preoperative symptoms and postoperative complications with each type proposed in the presents study. Abe et al. already reported that central lesions (corresponding with type 3) were more prone to preoperatively reveal a positive Tinel sign and numbness compared to peripheral lesions (corresponding with type 1) [17]. Moreover, postoperative work-up of the bPNST’s surgical specimen is going to include immunohistochemical methods so that each tumor can be allocated to a precise subtype [3]. Thus, any potential marker or characteristic might be identified that correlates with the types presented in this intraoperative morphological classification.

## 5. Conclusions

We were able to identify four different types regarding morphological relation between bPNST and healthy nerve fascicles. Our proposed classification might be used for preoperative high-resolution imaging and documentation of bPNST. Thus, it can aid in assessing the extent of informed consent for surgery, the surgical complexity, the need for nerve reconstruction and the risk for postoperative nerve morbidity according to the preoperative grading of the lesion.

## Figures and Tables

**Table 1:** Patient Demographics.

Parameter	*n*
Patients	31
Tumors	34
Age	48 ± 18
Sex (f/m)	20/11
Side (l/r)	16/18

**Table 2:** Morphological classification of bPNST based on their relation to healthy nerve fascicles presented via verbal description and schematic illustration. Schematic illustrations are presented in a side view (top) and cross-sectional view through the center of the lesion (bottom): healthy nerve fascicles are indicated in yellow color, while bPNST is displayed in pink and its capsule in grey. The epineural layer is shown in brown.

Type	Description	Scheme
Type I	bPNST is located peripherally	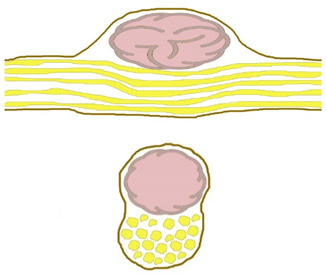
Type II	bPNST splits the nerve into 2 main fascicles	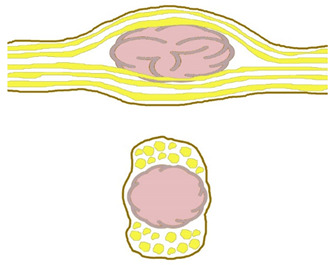
Type III	bPNST totally splits up the nerve out of the nerve’s center	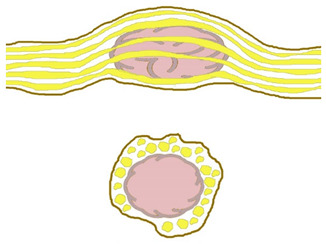
Type IV	bPNST encloses nerve fascicles without any detectable boundary layer	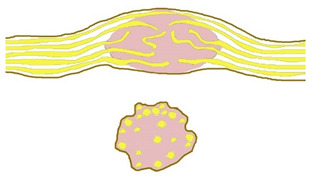

**Table 3:** Contingency table displaying number of cases and histological entities in every group.

	*n*	Schwannoma	Neurofibroma	Perineurioma
Type I	16	14	2	-
Type II	5	5	-	-
Type III	8	7	-	1
Type IV	5	2	3	-
Σ	34	28	5	1

## Data Availability

The data that support the findings of this study are available from the first author (M.H.) and the last author (M.S.), upon reasonable request.

## References

[1] Meyer A., Billings S.D. (2020). What’s new in nerve sheath tumors. Virchows Arch..

[2] Levi A.D., Ross A.L., Cuartas E., Qadir R., Temple H.T. (2010). The surgical management of symptomatic peripheral nerve sheath tumors. Neurosurgery.

[3] Rodriguez F.J., Folpe A.L., Giannini C., Perry A. (2012). Pathology of peripheral nerve sheath tumors: Diagnostic overview and update on selected diagnostic problems. Acta Neuropathol..

[4] Shanouda S., Kaya G. (2017). Benign Cutaneous Peripheral Nerve Sheath Tumor with Hybrid Features: Report of Two Cases with Schwannoma/Perineurioma and Schwannoma/Neurofibroma Components. Dermatopathology.

[5] Ronellenfitsch M.W., Harter P.N., Kirchner M., Heining C., Hutter B., Gieldon L., Schittenhelm J., Schuhmann M.U., Tatagiba M., Marquardt G. (2020). Targetable ERBB2 mutations identified in neurofibroma/schwannoma hybrid nerve sheath tumors. J. Clin. Investig..

[6] Louis D.N., Perry A., Reifenberger G., von Deimling A., Figarella-Branger D., Cavenee W.K., Ohgaki H., Wiestler O.D., Kleihues P., Ellison D.W. (2016). The 2016 World Health Organization Classification of Tumors of the Central Nervous System: A summary. Acta Neuropathol..

[7] Tamura R. (2021). Current Understanding of Neurofibromatosis Type 1, 2, and Schwannomatosis. Int. J. Mol. Sci..

[8] Powers C.J., Friedman A.H. (2007). A brief history of surgery for peripheral nerve sheath tumors. Neurosurg. Focus.

[9] Gosk J., Zimmer K., Rutowski R. (2004). Peripheral nerve tumours—Diagnostic and therapeutical basics. Folia Neuropathol..

[10] Knight D.M.A., Birch R., Pringle J. (2007). Benign solitary schwannomas: A review of 234 cases. J. Bone Jt. Surgery. Br. Vol..

[11] Kim D.H., Murovic J.A., Tiel R.L., Moes G., Kline D.G. (2005). A series of 397 peripheral neural sheath tumors: 30-year experience at Louisiana State University Health Sciences Center. J. Neurosurg..

[12] Russell S.M. (2007). Preserve the nerve: Microsurgical resection of peripheral nerve sheath tumors. Neurosurgery.

[13] Furniss D., Swan M.C., Morritt D.G., Lim J., Khanna T., Way B.L.M., Athanasou N.A., Giele H., Critchley P. (2008). A 10-year review of benign and malignant peripheral nerve sheath tumors in a single center: Clinical and radiographic features can help to differentiate benign from malignant lesions. Plast. Reconstr. Surg..

[14] Nilsson J., Sandberg K., Søe Nielsen N., Dahlin L.B. (2009). Magnetic resonance imaging of peripheral nerve tumours in the upper extremity. Scand. J. Plast. Reconstr. Surg. Hand Surg..

[15] Schmidt M., Kasprian G., Amann G., Duscher D., Aszmann O.C. (2015). Diffusion tensor tractography for the surgical management of peripheral nerve sheath tumors. Neurosurg. Focus.

[16] Pedro M.T., Antoniadis G., Scheuerle A., Pham M., Wirtz C.R., Koenig R.W. (2015). Intraoperative high-resolution ultrasound and contrast-enhanced ultrasound of peripheral nerve tumors and tumorlike lesions. Neurosurg. Focus.

[17] Abe K., Takeuchi A., Yamamoto N., Hayashi K., Tada K., Miwa S., Inatani H., Aoki Y., Higuchi T., Tsuchiya H. (2015). Symptomatic small schwannoma is a risk factor for surgical complications and correlates with difficulty of enucleation. Springerplus.

[18] Syme J. (1855). Lectures on clinical surgery: Lecture XXII Neuromata. Lancet.

[19] Harkin J.C., Reed R.J. (1969). Tumors of the Peripheral Nervous System.

[20] Stevens J., Davis D.H., MacCarty C.S. (1983). A 32-year experience with the surgical treatment of selected brachial plexus lesions with emphasis on its reconstruction. Surg. Neurol..

[21] Handler S.D., Canalis R.F., Jenkins H.A., Weiss A.J. (1977). Management of brachial plexus tumors. Arch. Otolaryngol..

[22] Donner T.R., Voorhies R.M., Kline D.G. (1994). Neural sheath tumors of major nerves. J. Neurosurg..

[23] Kline D.G., Judice D.J. (1983). Operative management of selected brachial plexus lesions. J. Neurosurg..

[24] Gosk J., Rutowski R., Zimmer K., Rabczyński J. (2004). Brachial plexus tumours--own experience in diagnostics and surgical treatment. Folia Neuropathol..

[25] Stone J.J., Spinner R.J. (2020). Go for the Gold: A “Plane” and Simple Technique for Resecting Benign Peripheral Nerve Sheath Tumors. Oper. Neurosurg..

[26] Tiel R., Kline D. (2004). Peripheral nerve tumors: Surgical principles, approaches, and techniques. Neurosurg. Clin. N. Am..

[27] Kwok K., Slimp J., Born D., Goodkin R., Kliot M., Berger M.S., Prados M.D. (2005). The evaluation and management of benign peripheral nerve tumors and masses. Textbook of Neuro-Oncology.

[28] Montano N., D’Alessandris Q.G., D’Ercole M., Lauretti L., Pallini R., Di Bonaventura R., La Rocca G., Bianchi F., Fernandez E. (2016). Tumors of the peripheral nervous system: Analysis of prognostic factors in a series with long-term follow-up and review of the literature. J. Neurosurg..

[29] Fujibuchi T., Miyawaki J., Kidani T., Miura H. (2017). Risk factors for neurological complications after operative treatment for schwannomas. J. Clin. Neurosci..

